# Long-Term Care Residents’ Priorities in Person-Centered Care: A Survey of Five Communities

**DOI:** 10.1093/geroni/igaf122.554

**Published:** 2025-12-31

**Authors:** Shih-Yin Lin, Elizabeth Seidel, Tara Cortes

**Affiliations:** New York University Rory Meyers College of Nursing, New York, New York, United States; New York University Rory Meyers College of Nursing, New York, New York, United States; New York University/HIGN, New York, New York, United States

## Abstract

As part of a multi-year project to establish person-centered care (PCC) standards for long-term care (LTC), this study explored how LTC residents perceive the importance of various PCC best practices and examined the influence of race/ethnicity and living type on their prioritization. A cross-sectional survey was conducted in five LTC communities across the U.S., where 470 residents (68.9% female; 66% non-Hispanic White, 25% non-Hispanic Black, and 9% Hispanic) rated the importance of 77 PCC best practices using a 5-point Likert-type scale. Descriptive statistics were calculated for resident characteristics and PCC ratings, and ANOVA was used to examine associations between demographics (race/ethnicity and living type) and PCC priorities. The top 10 ranked best practices focused on ‘Space and Living Environment’ (4 items), ‘My Health Care’ (3 items), ‘Communication with Staff’ (2 items), and ‘My Family, Friends, and Community’ (1 item). Findings revealed significant differences in PCC prioritization based on race/ethnicity and living type: Of the 77 best practices, Non-Hispanic White residents rated 53 items (69%) higher (p < 0.05) than non-Hispanic Black residents, and 10 items (13%) higher than Hispanic residents (p < 0.05). Independent living and skilled nursing residents rated all seven ‘Communication with Staff’ best practices higher than assisted living residents (p < 0.05). Additionally, independent living residents placed greater importance on ‘Participation in Leadership’ than both assisted living residents (p < 0.05 for 5 of the 7 items) and skilled nursing residents (p < 0.01 for all 7 items). These findings underscore the need to tailor PCC approaches based on resident race/ethnicity and living type.

